# Concomitant Central Giant Cell Granuloma and Aneurysmal Bone Cyst in a Young Child

**DOI:** 10.1155/2017/6545848

**Published:** 2017-04-05

**Authors:** Deepika Pai, Abhay Taranath Kamath, Adarsh Kudva, Monica Monica Charlotte Solomon, Saurabh Kumar, Prem Sasikumar

**Affiliations:** ^1^Department of Pedodontics & Preventive Dentistry, Manipal College of Dental Sciences, Manipal, India; ^2^Department of Oral and Maxillofacial Surgery, Manipal College of Dental Sciences, Manipal, India; ^3^Department of Oral Pathology, Manipal College of Dental Sciences, Manipal, India; ^4^Department of Oral and Maxillofacial Surgery, Mahe Institute of Dental Sciences, Puducherry, Kerala, India

## Abstract

Although Central Giant Cell Granuloma (CGCG) is a benign tumor of the jaw and aneurysmal bone cyst seen in children, its aggressive behavior causes extensive loss of hard tissue requiring wide excision and extensive rehabilitation. We report a rare case of concomitant CGCG and aneurysmal bone cyst in a two-year-old male child, involving the coronoid and condylar process. Young age, large tumor, its aggressive nature, and future growth of orofacial region pose a significant challenge in the management of such conditions. For a successful outcome, the systematic approach to the presurgical evaluation and appropriate treatment planning is essential for such conditions.

## 1. Introduction

Bony pathology in the region of head and neck in children is often seen as a morbid prognostic value. Since most of the lesions erode the jaws leading to disfigurement, or otherwise, the growth potential in children exaggerates the recurrence of the lesions.

Central Giant Cell Granuloma (CGCG) is a benign tumor of the jaw seen in children and young adults. CGCG accounts for approximately 7% of all benign tumors of the jaws; however, the aggressive nature of the lesion causes resorption of the tooth and bone in the area of the lesion leading to massive destruction of the bone, displacement of the erupted tooth, and germs of the unerupted tooth [1, 2]. It is more commonly seen in females and the mandible [3]. Based on the clinical behavior CGCG can be of either an aggressive or a nonaggressive variant. 30% of the cases are of aggressive variants and warrant wide excision of these lesions hence salvaging a large portion of the bone and tooth [3].

Aneurysmal bone cysts (ABC) are expansile osteolytic blood-filled cystic lesions seen commonly in the mandible [4]. Females are more often affected than males. It occurs with an age predilection of the first three decades of life. Depending on the extent and nature of the lesion, the treatment of ABC can vary from simple curettage to surgical resection [5, 6].

Wide excision of the aggressive lesion is the time-tested treatment modality in bony lesions of the jaw because of recurrence, but they usually disfigure the patients face. Rehabilitation following excision can also be challenging given the dynamic changes concerning growth of orofacial region occurring in the children. Hence the management of aggressive tumors of the jaw in children needs to be a continuous process with regular timed intervention keeping growth in the mind. The aim of this article is to report a rare case of concomitant CGCG and ABC in a two-year-old male child in the posterior region of the lower jaw.

## 2. Case Report

**Figure 1:**
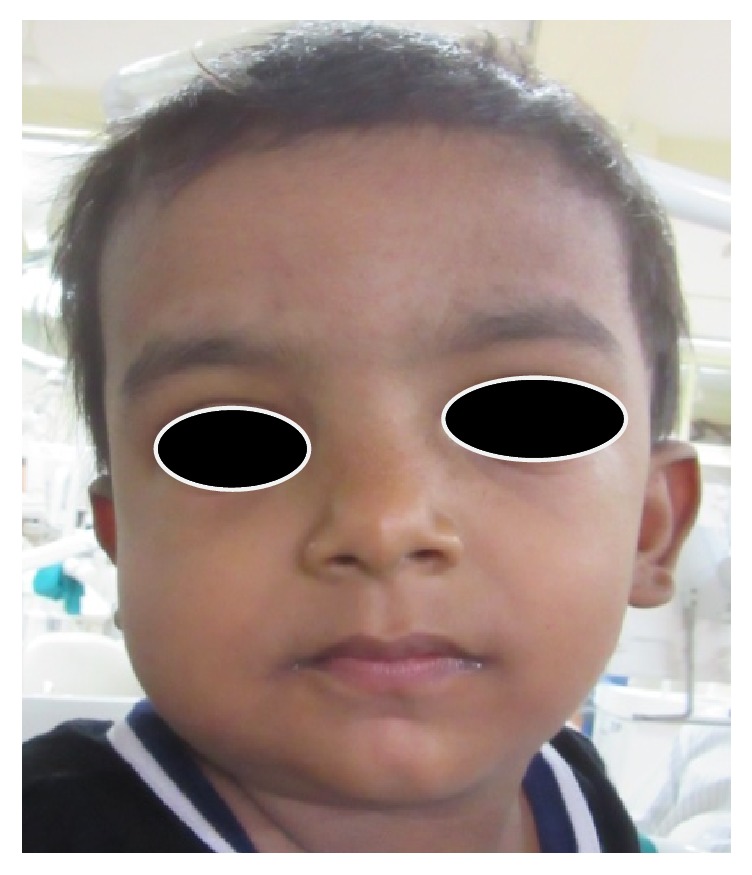
Swelling in relation to lower lateral side of face.

A two-year-old male child presented with a unilateral swelling on the right side of the face. The patient's mother noticed the swelling four months back, and since then the lesion had been progressively increasing in size. There was no history of trauma, trismus, fever, or familial history of a musculoskeletal disorder associated with the appearance of this swelling. On extraoral examination, the diffuse swelling was present with posterior region of the right side of the lower third of the face. Overlying skin was normal in color and texture with no signs of local inflammation (Figure 1). On palpation, the swelling was nontender, bony hard in consistency, and extending from the posterior border of the mandible to anterior margin of the ramus of the mandible. Crepitus and fluid thrills were absent. Paresthesia of lips was not present. Intraoral examination revealed erupted deciduous dentition with no carious lesion. The normal healthy intraoral mucosa was noted with no signs of local draining sinuses or periodontal pathology.

Laboratory investigations revealed normal serum values of calcium, phosphorus, urea, creatinine, and PTH. Alkaline phosphatase values were marginally raised. Screening radiographs for chest ribs and skull bones did not reveal the presence of such similar radiolucent lesions. On performing an ultrasonography, normal glandular echotexture and multiple benign upper deep cervical lymph nodes were noted bilaterally.

**Figure 2:**
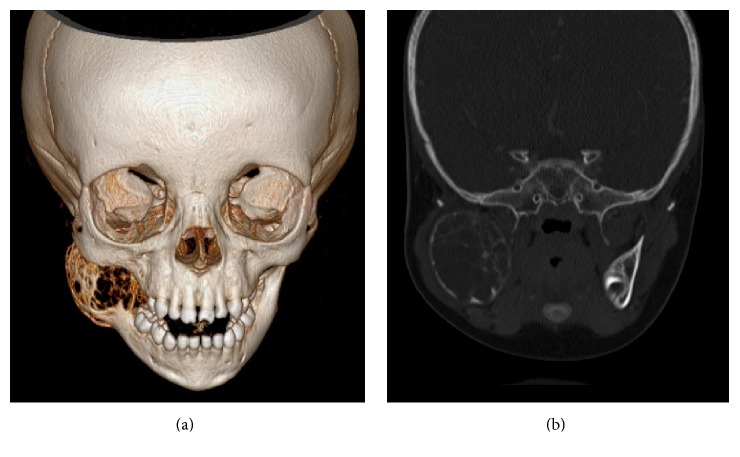
3D CT scan showing ballooning expansion of cortex.

Contrast enhanced CT scan showed large multiloculated expansive lesion measuring 4.2 cm anteroposteriorly, 3.5 cm mediolaterally, and 4 cm superoinferiorly, arising from right ramus of mandible extending to involve coronoid and condylar processes. Images showed uniform expansion resulting in the ballooning of the mandibular ramus (Figures 2(a) and 2(b)). The inferior alveolar nerve canal was displaced inferiorly. Follicle of the permanent first molar tooth was missing on the affected side as compared to normal side. Multiple sites of buccal, as well as lingual bony cortical plate perforation, were present. Overlying periosteal layer and adjacent tissue planes were intact and maintained anatomic continuity.

### 2.1. Histopathological Diagnosis

**Figure 3:**
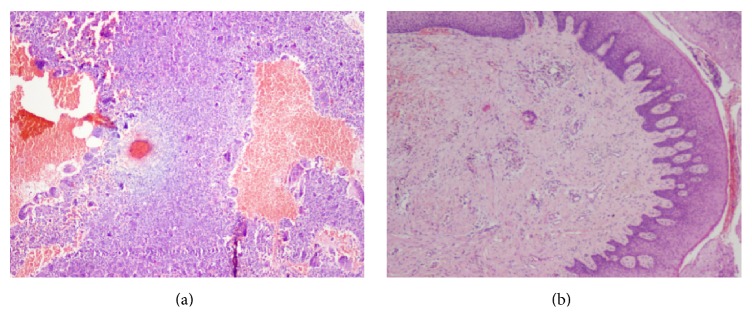
Microscopically the lesion is composed of blood-filled spaces separated by connective tissue septa containing fibroblasts, osteoclast-type giant cells.

The hematoxylin and eosin stain (H&E stain) section revealed a highly cellular stroma comprising abundant multinucleated giant cells, plump spindle cells, and stromal mononuclear cells. The multinucleated giant cells were diffusely distributed throughout the stroma with areas of hemorrhage, few chronic inflammatory cells, and many dilated RBC filled capillaries. Cysts-like spaces were also evident in certain areas which were lined by cellular connective tissue wall suggesting both Central Giant Cell Granuloma and aneurysmal bone cyst-like appearance (Figures 3(a) and 3(b)). Although multinucleate giant cells are a feature of the aneurysmal bone cyst and CGCG, the number of giant cells in this lesion is far beyond seen in aneurysmal bone cyst alone. The hemorrhagic areas were more closely associated with giant cells; that is why a diagnosis of CGCG and cyst-like areas was also evident; hence aneurysmal bone cyst-like features are also seen. Taking into consideration the histopathological observation, the clinical behavior of the lesion, and radiographic findings like the multilocular radiolucencies observed in our case the final diagnosis of concomitant CGCG and aneurysmal bone cyst was established [4, 5].

### 2.2. Treatment

With the histopathological diagnosis, the resection of right ramus condylar unit of mandible followed by reconstruction with costochondral grafting was planned. The surgical intervention includes curettage or wide excision depending on the nature and behavior of the lesion. The decision of resection was taken given the extensive bony involvement and multiple cortical bone perforation. In our case, since the lesion showed multiloculated radiographic appearance and was aggressive in nature surgical resection was planned over curettage to avoid recurrence.

**Figure 4:**
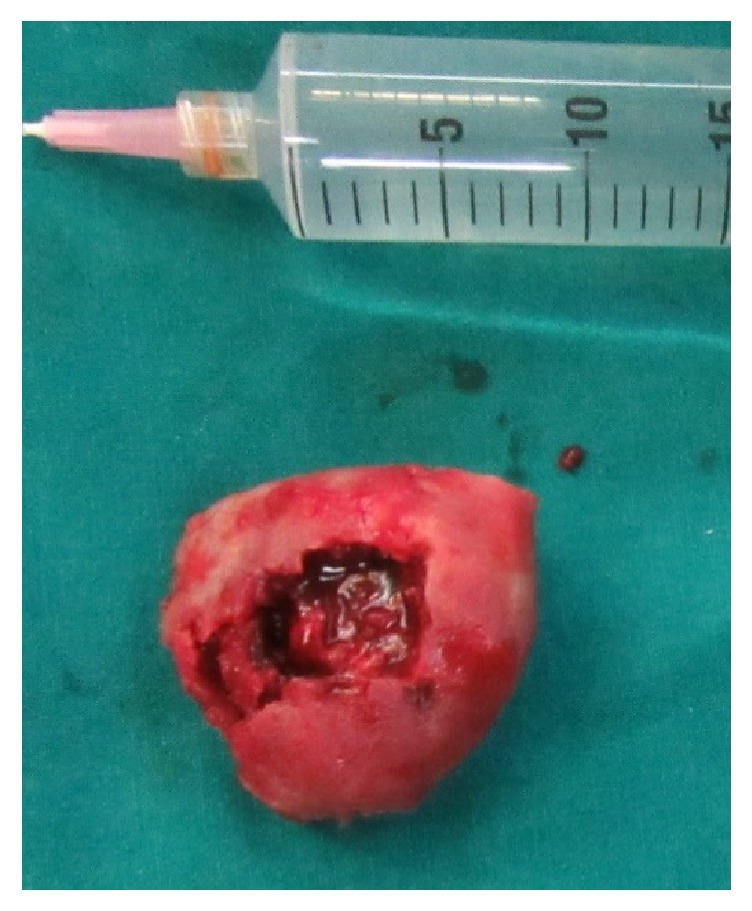
Intraoperative picture showing excised mass.

**Figure 5:**
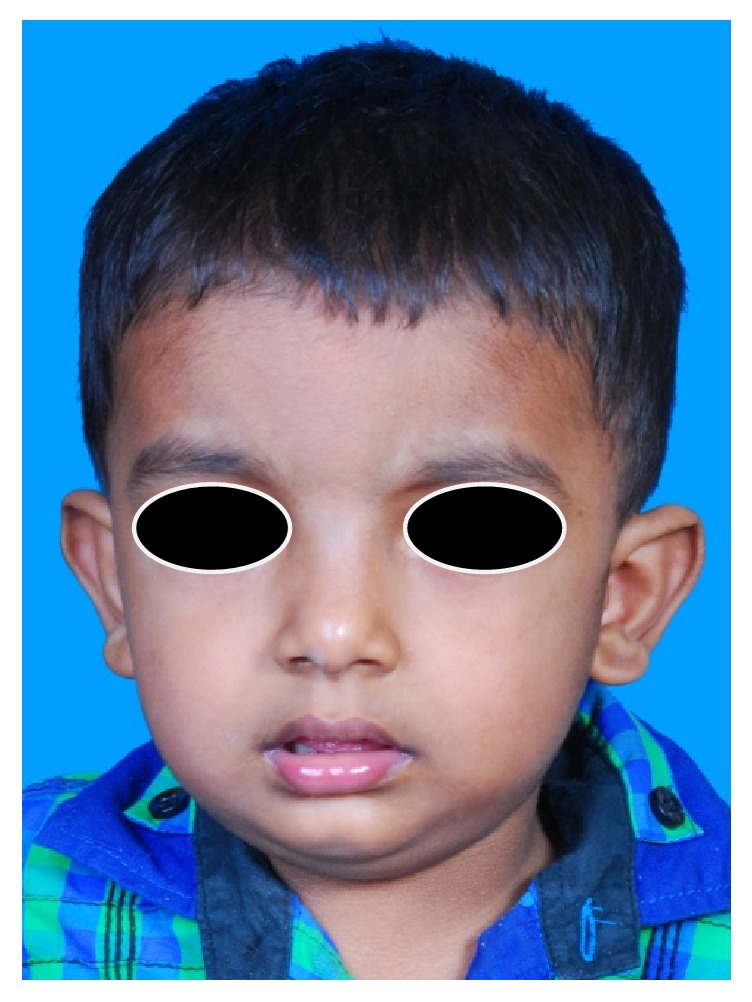
Postoperative photograph.

**Figure 6:**
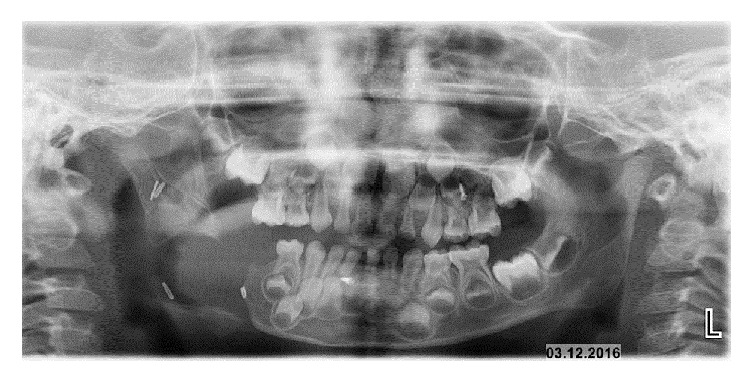
Postoperative OPG showing spontaneous regeneration of mandible.

A seven-centimeter-long extraoral submandibular incision was given along the neck crease. By layered sharp and blunt dissection, the inferior border of the mandible was exposed. Upon reflecting masseter and the periosteum, the surgically exposed bone showed ballooning and had a brownish hue. The inferior border of the angle of the mandible and ramus were involved. The bony lesion was resected, into healthy bony limits (Figure 4). Dark reddish or brownish granulation tissue was found, but it was not hemorrhagic. The excised lesion leads to loss of a large segment of the mandible which caused disfigurement of the face. Elastic intermaxillary fixation was done for guiding the mandible. The intermaxillary fixation is done in large lesions of a jaw that warrant wide excision. This facilitates the adequate establishment of occlusion postoperatively and helps in the appropriate orientation of the remaining jaw bone to the placement of the graft for rehabilitation. Postoperatively the patient was on follow-up for 18 months. The clinical appearance of the patient is excellent (Figure 5). Recent OPG revealed spontaneous regeneration of mandible (Figure 6).

## 3. Discussion

The behavior of the lesion is a critical factor in deriving a diagnosis of swelling seen in head and neck region in children. If one can categorize the swellings into groups based on the site of lesion, progression, and onset and duration of swelling, then a correct differential diagnosis can be obtained [7]. An acute swelling with inflammation and pain can be suggestive of lymphadenitis, odontogenic infection, skin abscess, and sinusitis. The differential diagnosis of nonprogressive swelling can be congenital anomalies like cephalocele, dermoid, and epidermoid cyst. Rapidly progressive facial swelling includes pediatric tumors like rhabdomyosarcoma, Langerhans cell histiocytosis, Ewing's sarcoma, osteogenic sarcoma, metastatic neuroblastoma, and similar lesions, whereas slowly progressive facial swelling as presented in our case includes neurofibroma, hemangioma, lymphangioma, vascular malformation, and fibroosseous lesions. Pathology of infective origin may also present as a slowly progressive swelling as in cases of Garre's osteomyelitis.

The fibroosseous diseases of the jaws represent a diverse group of entities. They commonly include lesions of primary or secondary hyperparathyroidism, fibrous dysplasia including cherubism, Central Giant Cell Granuloma, and aneurysmal bone cyst. The histopathological findings of these lesions may be remarkably similar, and hence they have to be differentially diagnosed by the clinical and radiographic presentation.

The clinical presentation of bony hard swelling with normal overlying skin color and normal cutaneous and subcutaneous echotexture on ultrasonography do not favor the diagnosis of neurofibroma, hemangioma, lymphangiomas, and vascular malformation. Certain intraosseous variants of vascular malformation and hemangiomas are reported; the radiographic presentation of multiloculated “soap bubble appearance” as seen in our case does not coincide with such intraosseous lesions of vascular origin. The absence of any intraoral infectious foci rules out osteomyelitis or similar chronic infectious lesions. The normal values of serum calcium, phosphorus, and alkaline phosphatase along with unilateral presentation indicate that the lesion was not cherubism or fibrous dysplasia.

Probable diagnosis of Central Giant Cell Granuloma (CGCG) can, therefore, be derived by sequentially ruling out another differential diagnosis. Radiographic “ballooning” of the ramus of mandible and “soap bubble appearance” further support the possible diagnosis. CGCG is predominantly found in children and young individuals but has not been commonly reported in a child as young as our patient. The coronoid and condylar process is rarely involved by the lesion [8]. The histopathologic examination in our case suggested the presence of both CGCG and ABC. Therefore the radiographic findings and clinical behavior of the lesion were also considered to derive upon a final diagnosis of concomitant CGCG and ABC.

The conventional treatment of lesions like CGCG or ABC of the jaw bones is surgical excision either by curettage or en bloc resection depending on the behavior (aggressive or nonaggressive), location, the size of the lesion, and radiographic appearance [9]. Other treatment options include drugs like Denosumab systemic injections of calcitonin and interferon and radiation.

When conservative options like Denosumab are used a complete response is rarely obtained; hence additional surgery becomes necessary to remove the tumor in case of tumor progression, to remove a remnant, or to remodel bone. Moreover, these drugs have frequent local or systemic side effects such as osteonecrosis and growth deficiencies [10–13].

Because of high recurrence rate of up to 70% after local curettage wide excision of the lesion is preferred as the choice of treatment [1, 14]. A review of various treatment options in patients with aggressive jaw lesions revealed that surgical resection is better than curettage as it can lead to undesirable damage to the jaw or teeth and tooth germs are often unavoidable, and recurrences are frequent [14–17]. Usually, surgical intervention in children is viewed with some skepticism. The uncertainty of intralesional corticosteroid injection and slow response to this kind of treatment, nature of lesion in our case being aggressive and multiloculated, and high recurrence rate of surgical modality like curettage led us to opt for surgical excision of the lesion.

The recent follow-up of our case revealed spontaneous regeneration of bone. It is an interesting finding to report since only a few reports exist on the spontaneous regeneration of bone after surgical resection of jaw lesions [18, 19]. It is found that such regeneration can significantly reduce or eliminate the need for reconstruction. This kind of regeneration is explained due to the presence of intact periosteum and its osteogenic potential in children. When the periosteum is preserved during surgical resection the new bone is generated that can fill the residual defect [18, 19]. The patient will continually be followed up for completion of regeneration of the defect and will be considered for further functional growth modification if any residual growth deformity exists.

## 4. Conclusion

Usually CGCG and ABC are seen with higher female predilection but in our case, it was seen in a very young boy and in unusual site with involvement of coronoid and condylar process. Proper histopathological examination and radiographic examination are much essential in establishing an accurate diagnosis, as in our case histopathological examination reported the presence of an aggressive case of concomitant CGCG and aneurysmal bone cyst. With this kind of unusual presentations, the clinician should always bear differential diagnosis in mind while examining bony swelling in head and neck region in children. With the evidence of spontaneous regeneration of resected portion of the mandible in our case, we suggest that when considering reconstructive options in such aggressive lesion of the jaw in children one must keep the host's growth potential in mind.
